# Highly Adaptable Analysis Tools for Mapping Spatial Features of Cellular Aggregates in Tissues

**DOI:** 10.1002/cpz1.70135

**Published:** 2025-05-05

**Authors:** Andrew Sawyer, Nick Weingaertner, Ellis Patrick, Carl G. Feng

**Affiliations:** ^1^ School of Medical Sciences, Faculty of Medicine and Health The University of Sydney Sydney NSW Australia; ^2^ Centenary Institute The University of Sydney Sydney NSW Australia; ^3^ Charles Perkins Centre The University of Sydney Sydney NSW Australia; ^4^ School of Mathematics and Statistics, Faculty of Science The University of Sydney Sydney NSW Australia; ^5^ The University of Sydney Institute for Infectious Diseases The University of Sydney Sydney NSW Australia

**Keywords:** cellular aggregates, granuloma, multiplex imaging, spatial analysis, tumor

## Abstract

Multiplex imaging technologies have developed rapidly over the past decades. The advancement of multiplex imaging has been driven in part by the recognition that the spatial organization of cells can represent important prognostic biomarkers and that simply studying the composition of cells in diseased tissue is often insufficient. There remains a lack of tools that can perform spatial analysis at the level of cellular aggregates (a common histopathological presentation) such as tumors and granulomas, with most analysis packages focusing on smaller regions of interest and potentially missing patterns in the overall lesion structure and cellular distribution. Here, we present protocols to quantitatively describe the cellular structure of entire tissue lesions, built around two novel metrics. The Total Cell Preference Index reports whether a lesion tends to change in density in its central versus peripheral areas and can indicate the extent of necrosis across the entire lesion. The Immune Cell Preference Index then reports whether each immune cell type is located more centrally or peripherally across the entire lesion. The output of both indexes is a single number readout for simple interpretation and visualization, and these indexes can be applied to lesions of any size or shape. This simplifies cross‐lesion comparison compared to traditional Euclidian distance–based analysis, which outputs multiple values for each lesion (one for output for each band used in the infiltration analysis). Additionally, this approach can be applied to any slide‐scanning multiplexed imaging system, based on either protein or nucleic acid staining. Finally, the approach uses the open‐source software QuPath and can be utilized by researchers with a basic understanding of QuPath, with the full analysis able to be applied to pre‐generated images within 1 hr. © 2025 The Author(s). Current Protocols published by Wiley Periodicals LLC.

**Basic Protocol 1**: Image preparation and lesion selection

**Basic Protocol 2**: Total Cell Preference Index and Immune Cell Preference Index

## INTRODUCTION

Multiplexed imaging technologies can now perform spatial profiling of dozens of epitopes across millions of cells, all in a single tissue section. These approaches generate enormous amounts of data and can map large, complicated tissue structures such as tumors and granulomas in their entirety within a sectional plane. It is increasingly being shown that the spatial organization of cells and necrotic tissue in these lesions can represent important prognostic biomarkers; however, most analytic tools focus only on cellular composition and/or small regions of interest within the lesions and miss trends at the level of the entire lesion. For example, the extent of leukocyte infiltration into solid tumors has been associated with better prognosis in many cancers (Grabovska et al., 2020; Pagès et al., 2010), whereas the development of necrosis and lack of cellularity in solid lesions are frequently associated with poorer prognosis (Liu & Jiao, 2019). As these platforms are able to resolve increasing numbers of cellular markers across larger sections of tissue, they are generating ever more complex datasets and can map tissue structures at high resolution in their entirety (Harms et al., 2023).

**Figure 1:**
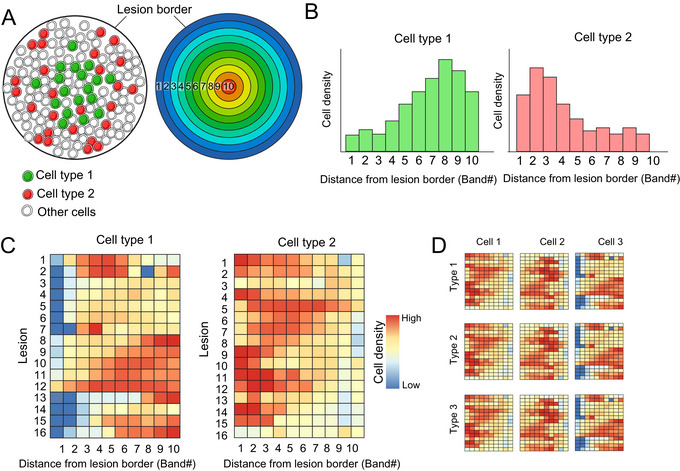
Band‐based infiltration analysis is impractical for quantifying intra‐lesion cell type distribution with high lesion throughput. (**A**) Schematic lesion images with cells colored according to the cell type (left) and with bands indicating regions in 10 increments from the lesion border (right). In both lesion images, the lesion border is indicated. (**B**) Example density histograms indicating the cell density of two cell types in 10 bands from the lesion border in a single image. (**C**) Example heatmaps indicating the cell density of two cell types in 10 bands from the lesion border from 16 lesions. (**D**) Example heatmaps to indicate the cell density of three cell types and lesions from three lesion types. Figure adapted from Sawyer et al. (2023).

Infiltration analysis is a common analysis method to measure the distance of cells from defined tissue lesions (Li et al., 2019; Page et al., 2023). Infiltration analysis requires the lesion border to first be annotated and is then typically performed using a series of software‐generated bands at fixed distances outward or inward from the lesion border (Fig. 1A). The density of the cells of interest is calculated in each of these bands to produce a density histogram to visualize the distribution of a cell of interest within a lesion (Attrill et al., 2022; Tunstall, 2016) (Fig. 1B). Although infiltration analysis is typically applied to study the relationship between leukocyte populations and a tumor, it can also be used to measure the distance from any cell population to any annotated tissue area.

This approach has proven useful for identifying novel biomarkers but has two key drawbacks. Firstly, it can be confounded when analyzing lesions of different sizes. For example, measuring cells within a 50‐µm bandwidth may have different relevance in a lesion of 100‐µm diameter compared to a lesion of 1000‐µm diameter, making cross‐lesion comparison difficult between lesions of different sizes. Secondly, when the information on multiple bands, cell types, and lesion types is included, the data produced by such analyses are challenging to visualize, as plotting the total results of an experiment is impractical, requiring an array of heatmaps to visualize the data (Fig. 1C and 1D).

**Figure 2:**
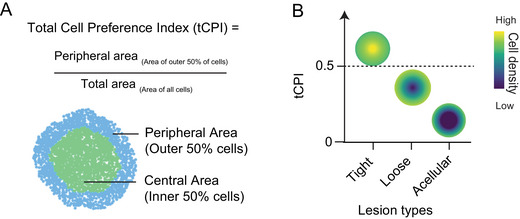
The tCPI is a single number measurement for intra‐lesion cell localization preference. (**A**) Lesion image with areas colored according to which cells are closer to or further from the lesion border relative to the median distance (bottom) and equation used to calculate the tCPI (bottom). (**B**) Schematic diagram illustrating the intra‐lesion cell distribution patterns and their corresponding tCPI values. Figure adapted from Sawyer et al. (2023).

Our set of protocols is designed to analyze multiplex images of entire lesions across the sectional plane. The two tools that this article centers on are the Total Cell Preference Index (tCPI) and the Immune Cell Preference Index (immCPI). The tCPI measures the densities of both the inner and outer 50% of cells in the lesion (relative to the lesion border) and expresses them as a ratio (Fig. 2A). This single number output can provide nuanced insight into how necrotic a lesion is and can give an indication of whether the lesion may be losing cells in the center and tending toward a necrotic phenotype while not actually being necrotic as of yet (Fig. 2B). Additionally, the tCPI tool can be applied to both immunofluorescence (IF) images and hematoxylin and eosin (H&E) staining images, as well as any other images that can be used to ascertain the spatial coordinates of each individual cell. The equation used to calculate the tCPI is as follows:

$$tCPI=\;\frac{Peripheral\;area_{\;\left(Area\;of\;outer\;50\%\;of\;cells\;in\;lesion\right)\;}}{\;Total\;area_{\;\left(Area\;of\;all\;cells\;in\;lesion\right)}}$$

**Figure 3:**
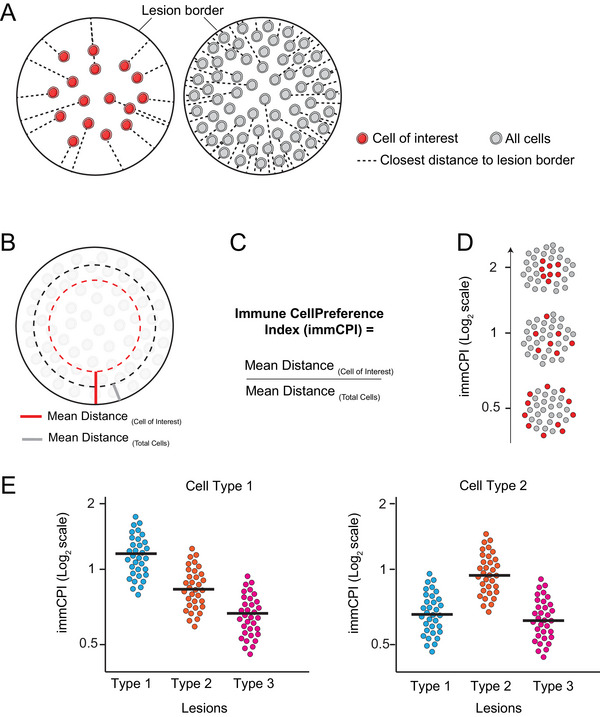
The immCPI quantifies the distribution of immune cell populations in any number of lesions and across lesion types. (**A**) Lesion images with only the cell of interest (red) visualized and the closest distance of each cell to the lesion border indicated with a dashed line (left) and with all cells (gray) illustrated and the closest distance of each cell to the lesion border indicated with a dashed line (right). (**B**) Schematic lesion image with the mean distance of both the cell of interest and all cells indicated by red and gray bands, respectively, within the lesion. (**C**) Equation used to calculate the immCPI. (**D**) Schematic diagram of lesions with representative immCPI measurements. Red, cell of interest; gray, the rest of the cells. (**E**) Example plots comparing the immCPI of two cell types between different lesion types. Figure adapted from Sawyer et al. (2023).

The immCPI measures the mean distance of all cells of each cell type from the lesion border and compares that with the mean distance of all cells in the lesion from the border (Fig. 3A to 3C). This provides a single number output indicating whether each cell type tends to localize more centrally or peripherally within the lesion (Fig. 3D). A single number output allows for simple visualization of the entire dataset with a single bar plot for each cell marker studied (Fig. 3E). Importantly, both these tools (tCPI and immCPI) can be applied to lesions of any size and shape. Additionally, these measures can be applied to any slide‐scanning multiplexed imaging system, based on either protein or nucleic acid staining. The equation used to calculate the immCPI is as follows:

$$immCPI=\;\frac{Mean\;distance\;from\;lesion\;border_{Cell\;of\;interest}}{Mean\;distance\;from\;lesion\;border_{All\;cells}}$$

The approach described here has previously been applied to the study of granulomas in the lungs of tuberculosis patients, which revealed a previously unknown level of lesion spatial heterogeneity (Sawyer et al., 2023). The approach is based on the open‐source software QuPath (Bankhead et al., 2017). It can be utilized by researchers with a basic understanding of QuPath, with the full analysis able to be applied to pre‐generated images within 1 hr. Overall, the approach used in this article provides a more nuanced picture of the spatial relationships between tissue lesions and cells than infiltration analysis using traditional Euclidian distance mapping. Tissue lesions in both cancers and infectious/inflammatory conditions like tuberculosis can be highly variable in their size and shape, which limits the extent to which inter‐lesion analysis can reliably be performed using Euclidian distance mapping. The tCPI and immCPI tools developed here enable rapid and simple cross‐lesion comparison that has the potential to continue to reveal novel biomarkers in tissue lesions.

## Basic Protocol 1 IMAGE PREPARATION AND LESION SELECTION

The purpose of this protocol is to identify and appropriately annotate lesions on which to perform tCPI and immCPI calculations (Basic Protocol 2). You will do this using basic functions of the software QuPath. Once completed, you should have accurately annotated all lesions in your tissue image.

### Materials

QuPath (Version 0.5.1)
Computer (a minimum RAM of 8 GB is preferable)
TIFF images generated from either H&E‐ or IF‐stained tissues
Computer mouse or graphics tablet

### Image preparation

1 Open QuPath on a computer (preferably with ≥8 GB RAM) and select “Create Project.”
2 Utilizing “Add images,” import TIFF images generated from either H&E‐ or IF‐stained tissues, with either whole slide scans or regions containing entire lesions, and select the appropriate scan type (Brightfield or Immunofluorescence).
3 Utilizing the contrast panel, change the channel min and max values to improve visibility of stained tissue.
  Changing values on the contrast panel will make things easier to see, but it will NOT change the underlying image used in analysis. For this reason, ascertaining the correct threshold values is critical at later stages.

### Lesion selection

4 In the “Annotations” tab, click the three vertical dots or right‐click the classifications region to expose a menu containing Add/Remove > Add Class. Create a class called “Tissue” and a class called “Lesion.”
5 Using the (P)olygon tool within QuPath and a computer mouse or graphics tablet, outline the border of the lesion.
6 Select and then right‐click the newly annotated lesion within the viewer and use “Set Classification” to select “Lesion.”
7 Similarly, outline the tissue and assign it the “Tissue” class.
8 Create a classification for each channel name or use the “Populate from Channels” function in the right‐click menu to do this in batch.
  It is possible to use thresholders as an alternative to automatically select the tissue based on the presence of a stain. This works particularly well with confluent stains such as eosin. For more information, visit the QuPath wiki at https://github.com/qupath/qupath/wiki.

## Basic Protocol 2 TOTAL CELL PREFERENCE INDEX AND IMMUNE CELL PREFERENCE INDEX

The purpose of this protocol is to apply two spatial metrics to the lesions identified in Basic Protocol 1. The tCPI and immCPI metrics are calculated using a single GROOVY script in QuPath. Code blocks are bounded by square brackets.

### Materials

See Basic Protocol 1.

1 Open the script editor, denoted by the </> icon in the toolbar.
2 Within the file menu, open the tCPI GROOVY script.
  The script is included at the conclusion of this protocol.
3 Alter the scanType variable such that it reflects the nature of the stain (IF or Brightfield):


[
scanType = "IF"
]

4 Within the If statements for the respective scanType, change the values of the watershed detection to reasonable values for the image being examined:


[
if(scanType=="Brightfield"){
runPlugin('qupath.imagej.detect.cells.WatershedCellDetection', '{"detectionImageBrightfield":"Hematoxylin OD","requestedPixelSizeMicrons":0.4994,
"backgroundRadiusMicrons":8.0,"backgroundByReconstruction":true,
"medianRadiusMicrons":0.0,"sigmaMicrons":1.4,"minAreaMicrons":5.0,
"maxAreaMicrons":400.0,"threshold":0.35,"maxBackground":2.0,
"watershedPostProcess":true,"cellExpansionMicrons":0.0,"includeNuclei":true,
"smoothBoundaries":true,"makeMeasurements":true}')
}
]

  Correct values for images depend entirely on the image being processed. To trial values, use the GUI controls available under Analyse > Cell Detection > Cell Detection menu, noting the values for the script.
  Alternative methods to detect cells including Stardist can be utilized instead by replacing the runPlugin command with the Stardist code block.
5 If immCPI calculation is being performed, set IncludeImmCPI variable to “Y” in order to run immCPI calculation for each marker defined in the header:


[
def IncludeImmCPI= "Y"
]

  The immCPI cannot be calculated based on brightfield imagery, as it requires cell type classifications to be made.
6 Optional: Set MarkerNames variable to include the exact names of the markers used for immCPI:


[
def MarkerNames = ["Opal 690","Opal 650","Opal 570", "Opal 540"];
]

7 Optional: Define classifiers in Classify > Object Classification > Create Single Measurement Classifier for each marker utilized. Make sure to give the classification the same name as the marker.
  When creating classifiers, set the channel filter to the appropriate marker, run for all detections in the object filter, and choose an appropriate measurement type for the cell (nucleus vs. cell; mean vs. min/max).
8 Optional: Create a composite classifier named “All Channels” in Classify > Object Classification > Create Composite Classifier containing all the markers.
9 Run the included script. Script also available at https://github.com/nwei0496/QuPathCPIScripts/.


[
/**
Script to determine the tCPI of an enclosed lesion. Script will detect cell objects within the defined region selected before calculating the median signed distance from lesion border and segmenting the area of the lesion into a central and peripheral median. The quotient of the peripheral area and the total area is defined as the total Central Preference
Index (tCPI) by Sawyer et al. (2023).
Code to create generate the infiltration annotation is inspired from code produced on the Image.sc Forum, particularly from Pete Bankhead. Regions of code where adapted script appears have been indicated in a comment block.
The script requires a lesion annotation with the "Lesion" class and an overall tissue annotation with class "Tissue".
Script will by default clear all detections when run. Ensure no detections are needed before running the script.
To preserve annotations change their class temporarily from "Lesion" so that they are not included in calculation of another lesion.
**/
import qupath.ext.stardist.StarDist2D
import qupath.lib.scripting.QP
import org.locationtech.jts.geom.Geometry
import qupath.lib.common.GeneralTools
import qupath.lib.objects.PathObject
import qupath.lib.objects.PathObjects
import qupath.lib.roi.GeometryTools
import qupath.lib.roi.ROIs
import java.awt.Rectangle
import java.awt.geom.Area
import org.locationtech.jts.precision.GeometryPrecisionReducer
import org.locationtech.jts.geom.PrecisionModel
import qupath.lib.gui.measure.ObservableMeasurementTableData
// ––––––––
// Things to modify
// ––––––––
// Do you want to keep inner annotation at end of script? Y/N
keepInnerAnnotation = "N"
// Is it Brightfield or IF
scanType = "IF"
// What are the exact names of the marker's used?
def MarkerNames = ["Opal 690","Opal 650","Opal 570", "Opal 540"];
// Do you want ImmCPI (Y/N)
def IncludeImmCPI= "Y"
// ––––––––
// End of Modification Block
// ––––––––
// Define ImmCPI Calculation
def ImmCpiCalc(ImmuneCellName, anno) {
def ImmuneCellDistance = [];
def RegularCellDistance = [];
getDetectionObjects().each{
if (it.getPathClass()!=null && it.getPathClass().toString().contains
(ImmuneCellName)) {ImmuneCellDistance.push(it.measurements.get("Signed distance to annotation with Lesion µm"))}}
if(ImmuneCellDistance.size()==0) {ImmuneCellDistance.push(0) }
getDetectionObjects().each{
RegularCellDistance.push(it.measurements.get("Signed distance to annotation with Lesion µm"))
} //print(ImmuneCellDistance)//try {// double ImmuneMeanDistance = ImmuneCellDistance.average().toDouble()//} catch(Exception e) {// double ImmuneMeanDistance = 0//}
double ImmuneMeanDistance = ImmuneCellDistance.average().toDouble()
double RegularMeanDistance = RegularCellDistance.average().toDouble()
double ImmCpi = ImmuneMeanDistance/RegularMeanDistance //ob.setImageData (imageData, tissuesTCPI);
def GranulomaObject = anno
GranulomaObject.measurements.put("ImmCPI("+ImmuneCellName+")",ImmCpi)
print("ImmuneCPI for "+ImmuneCellName+" is: "+ImmCpi)
}
// Define median function not in groovy by standard
def median(data) {
def copy = data.toSorted()
def middle = data.size().intdiv(2)
data.size() %2 ? copy[middle]: (copy[middle‐1] + copy[middle])/2
}
// Clear Detections
clearDetections()
selectObjectsByClassification("Lesion")
// Replace the following code blocks with settings for cell detection determined for particular tissue or with alternative models such as StarDist
if(scanType=="Brightfield"){
runPlugin('qupath.imagej.detect.cells.WatershedCellDetection', '{"detectionImageBrightfield":"Hematoxylin OD","requestedPixelSizeMicrons":0.4994,
"backgroundRadiusMicrons":8.0,"backgroundByReconstruction":true,
"medianRadiusMicrons":0.0,"sigmaMicrons":1.4,"minAreaMicrons":5.0,
"maxAreaMicrons":400.0,"threshold":0.35,"maxBackground":2.0,
"watershedPostProcess":true,"cellExpansionMicrons":0.0,"includeNuclei":true,
"smoothBoundaries":true,"makeMeasurements":true}')
}
if(scanType=="IF"){
runPlugin('qupath.imagej.detect.cells.WatershedCellDetection',
'{"detectionImage":"DAPI","requestedPixelSizeMicrons":0.25,
"backgroundRadiusMicrons":8.0,"backgroundByReconstruction":true,
"medianRadiusMicrons":0.0,"sigmaMicrons":1.5,"minAreaMicrons":8.0,
"maxAreaMicrons":500.0,"threshold":8,"watershedPostProcess":true,
"cellExpansionMicrons":3.0,"includeNuclei":true,"smoothBoundaries":true,
"makeMeasurements":true}');
if(IncludeImmCPI=="Y"){
runObjectClassifier("All Channels");
}}
//Measure distance from individual cell to lesion border
detectionToAnnotationDistancesSigned(true)
def signedDistances = [];
getDetectionObjects().each {
signedDistances.push(it.measurements.get("Signed distance to annotation with Lesion µm"))
}
medCellDistance = Math.abs(median(signedDistances)).toDouble()
print("Median distance of cells to lesion border is
"+medCellDistance.toString()+" µm")
PrecisionModel PM = new PrecisionModel(PrecisionModel.FIXED)
//Establish base env Variables
def imageData = getCurrentImageData()
def hierarchy = imageData.getHierarchy()
def server = imageData.getServer()
def cal = server.getPixelCalibration()
if (!cal.hasPixelSizeMicrons()){
print 'No pixel size calibration detected'
return
}
double expandPixels = medCellDistance / cal.getAveragedPixelSizeMicrons()
def initLesion = getAnnotationObjects().find{it.getPathClass() == getPathClass("Lesion")}
def lesionGeom = getAnnotationObjects().find{it.getPathClass()==getPathClass
("Lesion")}.getROI().getGeometry()
def plane = ImagePlane.getDefaultPlane()
def tissueGeom = getAnnotationObjects().find{it.getPathClass() == getPathClass("Tissue")}.getROI().getGeometry()
// Ensure lesion geometry is bound by tissue annotation. This covers casses where the lesion occurs on the edge of the tissue
lesionIntersectGeom = tissueGeom.intersection(lesionGeom)
lesionROIClean = GeometryTools.geometryToROI(lesionIntersectGeom, plane)
lesionIntersect = PathObjects.createAnnotationObject(lesionROIClean, getPathClass("Lesion"))
lesionIntersect.setName("Intersected Lesion")
generatedAnnotations = []
generatedAnnotations << lesionIntersect
/**
Following code block adapted from Pete Bankhead's script at
https://petebankhead.github.io/qupath/scripts/2018/08/08/three‐regions.html
**/
// Get the central area
def geomCentral = lesionIntersectGeom.buffer(‐expandPixels)
geomCentral = geomCentral.intersection(tissueGeom)
def roiCentral = GeometryTools.geometryToROI(geomCentral, plane)
def annotationCentral = PathObjects.createAnnotationObject(roiCentral)
annotationCentral.setName("Center")
// Get the inner margin area
def geomInner = lesionIntersectGeom
geomInner = geomInner.difference(geomCentral)
geomInner = geomInner.intersection(tissueGeom)
def roiInner = GeometryTools.geometryToROI(geomInner, plane)
def annotationInner = PathObjects.createAnnotationObject(roiInner)
annotationInner.setName("Inner margin")
periph = getPathClass("Periphery")
annotationInner.setPathClass(periph)
addObjects(annotationInner)
/**
–––––––
End code block
–––––––
**/
def regions = []
regions << initLesion
regions << annotationInner
def area = "Area µm^2"
def ob = new ObservableMeasurementTableData()
ob.setImageData(imageData,regions)
double lesionTotalArea = ob.getStringValue(initLesion, area).toDouble()
double peripheralArea = ob.getStringValue(annotationInner, area).toDouble()
double tCPI = peripheralArea/lesionTotalArea
initLesion.measurements.put("tCPI",tCPI)
print("tCPI is "+tCPI)
if(scanType=="IF" && IncludeImmCPI=="Y") {
for (marker in MarkerNames) {
ImmCpiCalc(marker,initLesion)
}
} if(keepInnerAnnotation=="N"){
removeObject(annotationInner, true)
}
]Code can also be accessed at https://github.com/nwei0496/QuPathCPIScripts/

10 Review the tCPI (and optional immCPI), which is printed to the console that runs the command as well as added to the measurement list for the annotation.

## COMMENTARY

### Background Information

Overall, this set of protocols provides a fast and user‐friendly approach for cross‐sample comparison of tissue lesions. Lesions of any size and shape can be interrogated using the tCPI and immCPI. The tCPI and immCPI outputs are both single number results to summarize the localization of large numbers of cells within lesions. This enables much easier cross‐lesion comparison than traditional Euclidian distance–based analysis that outputs multiple values for each lesion (one for output for each band used in the infiltration analysis). The effectiveness of traditional infiltration analysis is also limited when comparing lesions of different sizes (where bandwidths do not provide a 1:1 comparison), whereas both the tCPI and immCPI tools control for lesion size and can be reliably compared between any two lesions. Importantly, the tCPI and immCPI tools are not limited to use in QuPath or the protocol steps outlined here, as they can technically be applied in any image analysis platform that allows for the annotation of tissue lesions and measurement of cell distances from each annotation. For example, both the tCPI and immCPI can also be calculated in HALO, another widely used image analysis platform (Tunstall, 2016).

### Critical Parameters

Although these protocols can be applied to images generated with any multiplex imaging platform, it is highly advisable to use a tile‐based stitched imaging platform, such as the PhenoImager (Akoya) or PhenoCycler Fusion (Akoya), such that the largest possible tissue area can be imaged. This will provide the best chance of capturing lesions in their entirety and has the potential of capturing multiple lesions (potentially dozens from a single slide when lesions are small). Images of large areas of tissue with highly multiplexed IF staining may also require greater computing power for smooth viewing and annotation.

It is important that a reliable method of segmenting individual cells is available, as both the tCPI and immCPI rely on the spatial locations of each cell centroid. Typically, this will require the addition of a nuclear dye, such as DAPI, to label each cell. It is important to ensure DAPI staining is clear, as poor‐quality nuclear staining may lead to inaccurate cellular detection.

Both the tCPI and immCPI tools also require an annotation of the lesion's outer border, which can sometimes be difficult. This can be achieved via multiple means and will differ depending on the disease being studied, which cellular markers are available, and whether the image is immunohistochemistry (IHC) or IF based. In tumors, this is often achieved by the identification of a tumor‐specific marker, such as SOX10 in the case of melanomas (Bahmad et al., 2023). In the case of tuberculosis lesions, these have been identified through a combination of H&E staining and high‐density aggregates of leukocytes in IF images.

Finally, whereas the tCPI tool can be applied to both H&E and IF images, the immCPI tool is only applicable to IF images. The tCPI only requires cell segmentation to have been performed, which is possible in H&E images by using hematoxylin staining; however, the immCPI requires the cellular identity of each leukocyte population to have been elucidated, which is not possible in H&E images by scripting in QuPath.

### Troubleshooting

Please see Table 1 for a troubleshooting guide outlining common problems and their solutions.

**Table 1:** Troubleshooting Guide for Mapping Spatial Features of Cellular Aggregates in Tissues

Problem	Possible cause	Solution
Lesion border is not able to be clearly delineated (related to Basic Protocol 1)	Some immune lesions (for example, in the case of tuberculosis) do not have a clear lesion border to set the annotation and use as an anchor for the tCPI and immCPI measurements	Perform imaging of H&E‐ and IF‐stained consecutive serial tissue slides (with major immune populations such as macrophages, T cells, etc.). Cross‐referencing can give a clearer picture of where the edge of the tissue lesion lies.
		Try to draw the lesion border as close to the edge as possible. Do not attempt to draw a border larger than the lesion (to try and make sure you capture the whole lesion) or smaller than the lesion (to ensure you only capture the lesion area), as performing either of these actions will compromise the immCPI and tCPI measurements.
Lesion has a highly irregular shape or no clear center (related to Basic Protocol 1)	Circular lesions represent the simplest overall structure, but in some cases, lesions may have branching or multifocal shapes that do not have a clearly visible center	As long as the lesion has a continuous (non‐broken) structure, both the tCPI and immCPI tools will still function on the lesion. The lesion should be considered as a single lesion regardless of the size or complexity of the shape.

### Understanding Results

The tCPI metric outputs a single number result between 0 and 1 for the analysis of each lesion. A result of 0.5 indicates that the inner 50% of cells in a lesion have the same density as the outer 50% of cells from the lesion border and implies that the lesion has a consistent cell density and is unlikely to be necrotic or tending toward a necrotic phenotype. A result above 0.5 indicates that the inner 50% of cells have a higher density compared to the average density of the lesion and implies that the lesion has a tight aggregation of cells in its core. Finally, a score below 0.5 indicates a lesion with a density of the inner 50% of cells less than the average density of the lesion. A score between 0.4 and 0.5 implies that the lesion has a slightly reduced density in its central region and may tend toward a necrotic phenotype; however, the lesion may not necessarily having the pathological appearance of a necrotic lesion. A score below 0.4 typically indicates a lesion with pathological necrosis, and the closer to 0 the tCPI value, the greater the acellular region within the lesion.

**Figure 4:**
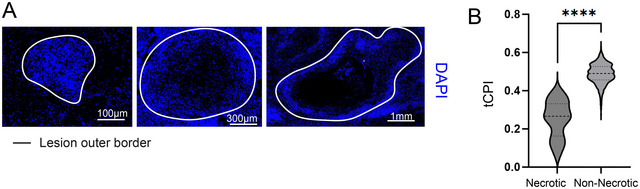
Cells in non‐necrotizing lesions show diverse spatial distribution preferences. (**A**) Images of DAPI‐stained tissue showing variations in intra‐lesion cell distribution. (**B**) tCPI values compared between necrotizing and non‐necrotizing lesions. The level of statistical significance was determined using a Mann‐Whitney test, and *p* < .05 was considered to indicate statistical significance. ****p < .0001. Figure adapted from Sawyer et al. (2023).

We have previously applied the tCPI tool to the study of 672 tuberculosis lesions from the lungs of 13 patients (Sawyer et al., 2023). At the level of segmented DAPI‐positive cells, we observed that there appeared to be a high diversity in the tightness and looseness of lesion structures, and we identified a spectrum of lesions ranging from tight cellular aggregates to loose, potentially pre‐necrotic lesions to large necrotizing granulomas (Fig. 4A). Using the tCPI tool, we were able to verify that pathologically confirmed necrotizing granulomas had the lowest tCPI score, whereas pathologically non‐necrotizing lesions’ scores ranged from 0.6 to 0.3 (Fig. 4B).

The immCPI metric outputs a single number result, usually between 0.5 and 2. An immCPI score of 1 for a cell type of interest within a lesion implies that the mean distance of that cell from the lesion border is equal to the mean distance of all cells in the lesion from the lesion border and indicates that the cell type is evenly distributed between the lesion periphery and the lesion core. An immCPI value less than 1 implies that the cell of interest is distributed more peripherally in the lesion compared to the mean distance of all cells from the lesion border, whereas an immCPI value greater than 1 implies that the cell of interest is distributed more centrally in the lesion. Importantly, the immCPI tool can provide a comparable output for lesions regardless of whether any of the lesions have necrotic cores, as the immCPI is normalized to all cells in a lesion and automatically excludes necrotic areas from the calculation.

**Figure 5:**
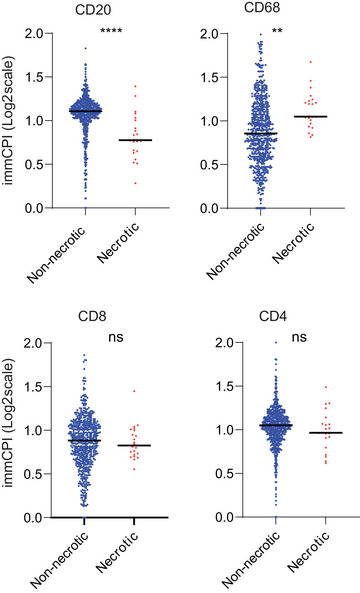
Example showing divergent intra‐lesion spatial distribution of CD20+ cells and CD68+ cells between necrotic and non‐necrotic tuberculosis granulomas. The immCPI of each immune cell type across tuberculosis lesion types is shown. Each symbol represents an individual lesion. Solid black lines indicate the group mean. The level of statistical significance was determined with a Mann‐Witney test, and *p* < .05 was considered to indicate statistical significance. **p < .01. ****p < .0001. ns, Not significant. Figure adapted from Sawyer et al. (2023).

We have previously applied the immCPI tool to the prior cohort of tuberculosis lesions following the identification of four major cell populations based on IF staining and five classes of lesions based on cell composition and lesion tCPI (Sawyer et al., 2023). We compared the immCPI of each cell type in each lesion across the five lesion categories. The immCPI tool revealed prominent differences in the preference of each cell type to localize centrally in each lesion class and allowed simple visualization of all parameters of the analysis (Fig. 5).

Here, we provide a workflow for analysis of images from solid tissue lesions generated from any imaging platform. We demonstrate that this workflow can identify the structural characteristics of tuberculosis lesions as an example. This workflow provides a reliable methodology for the analysis of entire cell aggregates and is readily adaptable to future development of imaging platforms to capture tissue lesions in increasing detail.

### Time Considerations

Basic Protocol 1 can be completed by someone with a basic knowledge of QuPath in ∼30 min for a tissue slide containing a dozen lesions.

Basic Protocol 2 can be completed in 5 min.

### Author Contributions

**Andrew Sawyer**: Conceptualization; investigation; visualization; writing—original draft; writing—review and editing. **Nick Weingaertner**: Methodology; software; writing—review and editing. **Ellis Patrick**: Formal analysis; methodology; software. **Carl G. Feng**: Conceptualization; funding acquisition; supervision; writing—review and editing.

### Conflict of Interest

The authors declare no conflicts of interest.

## Data Availability

Data sharing is not applicable to this article as no new data were created or analyzed in this study.
